# Software for Administering the National Cancer Institute’s Patient-Reported Outcomes Version of the Common Terminology Criteria for Adverse Events: Usability Study

**DOI:** 10.2196/10070

**Published:** 2018-07-16

**Authors:** Martin W Schoen, Ethan Basch, Lori L Hudson, Arlene E Chung, Tito R Mendoza, Sandra A Mitchell, Diane St. Germain, Paul Baumgartner, Laura Sit, Lauren J Rogak, Marwan Shouery, Eve Shalley, Bryce B Reeve, Maria R Fawzy, Nrupen A Bhavsar, Charles Cleeland, Deborah Schrag, Amylou C Dueck, Amy P Abernethy

**Affiliations:** ^1^ Division of Hematology and Medical Oncology Department of Internal Medicine Saint Louis University School of Medicine Saint Louis, MO United States; ^2^ Division of Hematology/Oncology Department of Medicine University of North Carolina School of Medicine Chapel Hill, NC United States; ^3^ Department of Epidemiology and Biostatistics Memorial Sloan Kettering Cancer Center New York, NY United States; ^4^ Lineberger Comprehensive Cancer Center University of North Carolina Chapel Hill, NC United States; ^5^ Department of Health Policy and Management Gillings School of Public Health University of North Carolina Chapel Hill, NC United States; ^6^ Duke Clinical Research Institute Duke University Durham, NC United States; ^7^ Division of General Medicine and Clinical Epidemiology Department of Medicine University of North Carolina School of Medicine Chapel Hill, NC United States; ^8^ Division of General Pediatrics & Adolescent Medicine Department of Pediatrics, Program on Health & Clinical Informatics University of North Carolina School of Medicine Chapel Hill, NC United States; ^9^ Department of Symptom Research The University of Texas MD Anderson Cancer Center Houston, TX United States; ^10^ Division of Cancer Control and Population Sciences National Cancer Institute Rockville, MD United States; ^11^ Division of Cancer Prevention National Cancer Institute Rockville, MD United States; ^12^ SemanticBits Herndon, VA United States; ^13^ Center for Biomedical Informatics and Information Technology National Cancer Institute Rockville, MD United States; ^14^ Duke Cancer Institute Durham, NC United States; ^15^ FHI 360 Durham, NC United States; ^16^ Division of General Internal Medicine Duke University School of Medicine Durham, NC United States; ^17^ Division of Population Sciences Dana-Farber Cancer Institute Boston, MA United States; ^18^ Alliance Statistics and Data Center Mayo Clinic Scottsdale, AZ United States; ^19^ Flatiron Health New York, NY United States

**Keywords:** usability, patient-reported outcomes, symptoms, adverse events, PRO-CTCAE, cancer clinical trials

## Abstract

**Background:**

The US National Cancer Institute (NCI) developed software to gather symptomatic adverse events directly from patients participating in clinical trials. The software administers surveys to patients using items from the Patient-Reported Outcomes version of the Common Terminology Criteria for Adverse Events (PRO-CTCAE) through Web-based or automated telephone interfaces and facilitates the management of survey administration and the resultant data by professionals (clinicians and research associates).

**Objective:**

The purpose of this study was to iteratively evaluate and improve the usability of the PRO-CTCAE software.

**Methods:**

Heuristic evaluation of the software functionality was followed by semiscripted, think-aloud protocols in two consecutive rounds of usability testing among patients with cancer, clinicians, and research associates at 3 cancer centers. We conducted testing with patients both in clinics and at home (remotely) for both Web-based and telephone interfaces. Furthermore, we refined the software between rounds and retested.

**Results:**

Heuristic evaluation identified deviations from the best practices across 10 standardized categories, which informed initial software improvement. Subsequently, we conducted user-based testing among 169 patients and 47 professionals. Software modifications between rounds addressed identified issues, including difficulty using radio buttons, absence of survey progress indicators, and login problems (for patients) as well as scheduling of patient surveys (for professionals). The initial System Usability Scale (SUS) score for the patient Web-based interface was 86 and 82 (*P*=.22) before and after modifications, respectively, whereas the task completion score was 4.47, which improved to 4.58 (*P*=.39) after modifications. Following modifications for professional users, the SUS scores improved from 71 to 75 (*P*=.47), and the mean task performance improved significantly (4.40 vs 4.02; *P*=.001).

**Conclusions:**

Software modifications, informed by rigorous assessment, rendered a usable system, which is currently used in multiple NCI-sponsored multicenter cancer clinical trials.

**Trial Registration:**

ClinicalTrials.gov NCT01031641; https://clinicaltrials.gov/ct2/show/NCT01031641 (Archived by WebCite at http://www.webcitation.org/708hTjlTl)

## Introduction

Symptomatic adverse events (AEs) such as nausea and fatigue are common in cancer clinical trials [1]. Historically, this information has been reported by clinicians using the National Cancer Institute (NCI) Common Terminology Criteria for Adverse Events (CTCAE), the most commonly used system for AE reporting [2]. To enable patients to directly report this information, the NCI recently developed the Patient-Reported Outcomes version of the CTCAE (PRO-CTCAE) item library as a companion to CTCAE. PRO-CTCAE includes 78 symptomatic AEs; for each symptomatic AE, 1-3 distinct items are used to evaluate the presence, frequency, severity, and associated interference with usual or daily activities for a total of 124 items [3]. PRO-CTCAE is designed to be administered frequently in trials, for example, weekly, and it records the worst magnitude for severity assessment, in accordance with the tenets of AE reporting. These AEs can be individually elicited and are not aggregated into global scores compared with other reporting methods. Development and testing of PRO-CTCAE items, including validity, reliability, responsiveness, mode equivalence, and recall period, have been previously reported [4-6].

As part of the development of the PRO-CTCAE items, prototype software was developed [7]. The key functionalities were derived from an iterative process, including patients, clinical trialists, administrators, NCI, and Food and Drug Administration stakeholders, and included the following:

1. *Professional (clinician and research associate) interface*: This includes a form builder that enables selection of PRO-CTCAE items and a configurable alert system that activates emails if patients miss a scheduled self-report or patients self-report a severe or worsening AE. Additionally, it includes tools for displaying patient-reported information with various levels of access restriction, given the use of the software by different user types.
2. *Survey scheduling*: A graphical calendar that enables scheduling or timing of patient survey administration, which is configurable by study and has the ability to shift dates in real time at the patient or study level if treatment schedules are modified during a given trial.
3. *Patient survey interface*: Surveys are administered to patients through a Web-based survey that presents questions for each AE together on a page (based on prior research) [8] or an automated telephone interactive voice response (IVR) system. “Conditional branching” is included for AEs with more than one question and a free text box is included at the end for patients to add additional symptoms via dropdown options or to enter unstructured text.

Creating such a system is complex, given the necessity for considerations around security and privacy, diverse computer literacy levels of patients, the need to integrate PRO data into the workflow of professionals, and required compliance with US government Section 508 specifications to ensure that the software was accessible to users with disabilities [9]. Thus, before scaling the system for large-scale implementation in clinical trials, we sought to optimize its usability by testing with end users (patients and clinical trial staff). We have described the usability assessment of the PRO-CTCAE system with a combination of evaluation methods in order to facilitate future adoption of the system into oncology research efforts [10] and improve clinical data collection and patient safety [9].

The aims of this study were (1) to perform a heuristic evaluation of the software to determine functionality problems, deviations from best practice, and compliance with regulations; (2) to conduct Round 1 of the initial usability testing using both quantitative and qualitative methods with target users, patients with cancer, and professionals that treat cancer; and (3) to refine the PRO-CTCAE system with software development and re-evaluate its usability with Round 2 of testing and include remote testing and IVR system evaluation.

## Methods

### Study Approach

**Figure 1:**
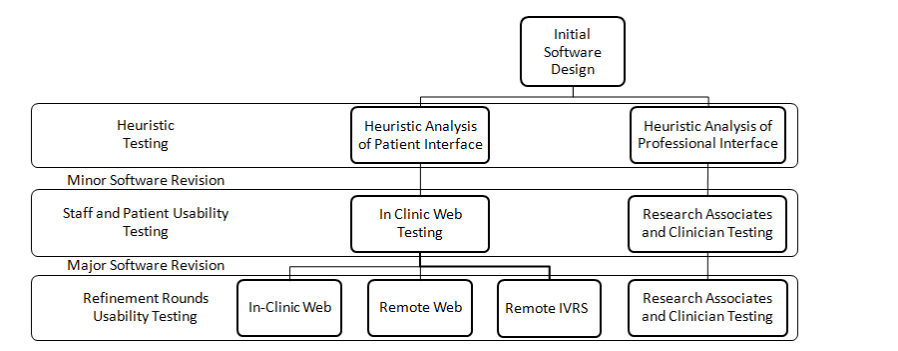
Study description and usability testing plan. IVRS: interactive voice response system (automated telephone).

A protocol for usability testing was approved by the institutional review boards at the NCI and 3 participating institutions, Duke University (Durham, NC), MD Anderson Cancer Center (Houston, TX), and Memorial Sloan Kettering Cancer Center (New York, NY). The study approach to test and refine the PRO-CTCAE software consisted of two interrelated components, heuristic evaluation, followed by successive rounds of iterative user-based usability testing (Figure 1), to interrogate the following discrete, well-established domains [10,11]: ease of learning, efficiency of use, memorability, error frequency and severity, and subjective satisfaction (Multimedia Appendix 1) [12].

#### Aim 1: Heuristic Evaluation

Heuristic evaluation is an inspection method that identifies usability problems through examination to evaluate compliance with recognized principles [13]. Usability experts interacted with the system and performed all tasks involved in creating and completing a survey to identify common issues related to collection and communication of PRO-CTCAE data for cancer clinical trials [14]. Usability heuristics were applied to all tasks of both patient and professional users to facilitate patient symptom reporting [15]. Results were organized into heuristic categories and discussed by the research team in order to develop solutions, guide software modifications, and identify potential challenges prior to user-based testing [16].

#### Aims 2 and 3: User-Based Usability Testing

User-based testing involves observation of end users to evaluate the ease of navigation, interaction with application features, ability to perform essential functions, and satisfaction with task flow [17]. We performed user-based testing of the PRO-CTCAE software with patients receiving systemic cancer treatment and among professional users (physicians, nurses, and research associates). We obtained informed consent from all users for participation in this study.

The usability investigative team (represented by the authors) analyzed the PRO-CTCAE software core functionalities and identified key tasks for testing [18]. The performance of these tasks by end users was directed by experienced evaluators using semiscripted guides that incorporated the “think-aloud” method [19] (see Multimedia Appendix 1 and Patient and Professional Protocols in Multimedia Appendix 2). Evaluators monitored how test subjects interacted with the system, while users were concurrently asked to describe their thoughts and actions during which comments were documented. These comments were categorized into usability problem types and classified as positive, neutral, or negative [20]. We flagged all comments that contained suggestions for improvements for review. Furthermore, a “task completion” scale ranging from 0 to 5 was developed to gauge the difficulty of each usability task (Table 1). After testing, all users completed the System Usability Scale (SUS) that evaluated the usability from 0 to 100, with high scores indicating high usability and scores above 68 indicating better than average usability [21,22].

Consistency among evaluators at each site was emphasized during on-site training conducted by experienced usability evaluators (MS and LH) and was supplemented by subsequent remote booster training. To capture the evaluations of professional staff, evaluators followed a semiscripted guide that was based on prior analysis of key system functions [23]. Accordingly, two rounds of testing were planned, with a targeted sample size of 40-65 professionals (physicians, nurses, and research associates) and 160-195 patients. Based on the conceptual saturation of usability testing issues, the study design included an option to add a third round if usability issues were not resolved through refinement between the first two rounds of testing.

**Table 1:** Quantitative task completion.

Assistance and score		Score description
**Assistance not necessary**		
	5	Completed task easily
	4	Task performed with hesitation or single error
	3	Achieved task with confusion or with multiple inappropriate clicks
**Assistance provided**		
	2	Completed with single prompt
	1	Task performed after multiple prompts and help
	0	Despite prompts, task not completed correctly

The investigative team, in collaboration with human factors consultants, reviewed the results in Round 1 to create solutions and software revision priorities to address the identified limitations in functionality and usability. Subsequently, we tested these modifications in Round 2 and again reviewed to determine if issues were satisfactorily resolved or if further revision or testing was warranted. “Usability” was predefined as the presence of a ceiling effect in the performance measurement and resolution of all identified significant problems amenable to software innovation [19].

#### Patient Testing

Patient testing focused on the completion of PRO-CTCAE questions using two different available data entry interfaces, Web-based interface and IVR system. We approached patients receiving outpatient systemic cancer treatment in clinic waiting rooms and invited them to participate in this study if they could speak English and did not have cognitive impairment that would have precluded the understanding of informed consent and meaningful participation in a usability testing. An accrual enrichment strategy was employed to oversample for participants who were ethnically and racially diverse, had high school education or less, were aged >65 years, and had limited baseline computer experience. The accrual of participants with these characteristics was monitoring during weekly calls; we discussed strategies for recruiting and enrolling patients with these characteristics.

In Round 1, all participants were asked to perform a series of scripted tasks (eg, log in the system, answer survey questions, and add a symptom), while being observed in private areas of clinic waiting rooms. Evaluators took notes regarding user responses to scripted tasks and questions and audiorecorded the interactions for subsequent transcription and analysis.

In Round 2, patient participants were asked to complete a series of PRO-CTCAE tasks while being monitored in the clinic or remotely without assistance or supervision. For remote testing, patient participants were assigned either to use the Web-based interface or IVR system. Instructions for using these interfaces were provided on an information card with login instructions, and an instructional video was also available. After the remote completion of the PRO-CTCAE tasks, an evaluator contacted each participant and asked semiscripted questions about the usability that focused on ease-of-use and difficulties associated with each task. Remote use was emphasized in Round 2 because it was anticipated that many future trial participants would be accessing the PRO-CTCAE software from home and would not have staff available to assist.

#### Physicians, Nurses, and Research Associates Testing

The evaluators observed the users as they completed a scripted series of tasks and audiorecorded encounters for transcription and analysis. In Round 1, the testing was evenly distributed among professional roles, whereas in Round 2, the testing focused predominantly on research associates, as it was anticipated that they would perform a majority of tasks associated with scheduling and processing of PRO-CTCAE data during trials.

### Study Sites

We enrolled all participants from 3 academic cancer hospital outpatient clinics and their affiliated community oncology practices (Duke University, MD Anderson Cancer Center, and Memorial Sloan Kettering Cancer Center). Recruitment was monitored weekly to ensure that the accrual was on schedule and enrichment procedures were being followed and to reinforce consistency of study methods.

### Statistical Analysis

All data were entered into REDCap version 4 (Vanderbilt University, Nashville, TN) SPSS version 21 (IBM, Armonk, NY) was used for analyses. For each usability task, we compared the mean task completion score between each round using independent sample *t* tests and compared them with other tasks in the same round using repeated measures analysis of variance (ANOVA). We performed pairwise comparisons following ANOVA using the Tukey’s Honest Significant Difference test. Furthermore, all statistical tests were two-sided, and we considered *P*<.05 as statistically significant.

## Results

### Aim 1: Heuristic Evaluation

**Figure 2:**
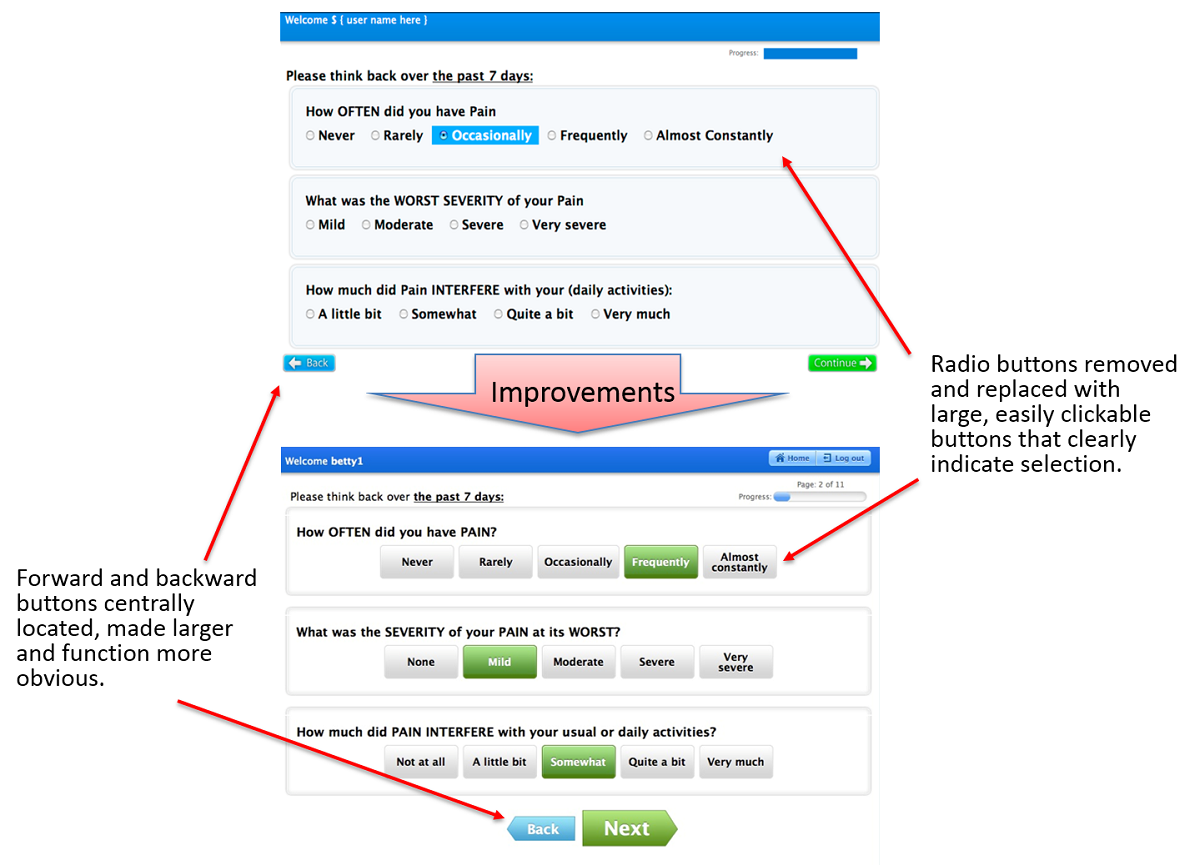
Screenshots of the Patient-Reported Outcomes version of the Common Terminology Criteria for Adverse Events (PRO-CTCAE) before and after usability improvements to benefit end-users: patient.

The system was inspected by 2 usability experts using established heuristics to identify usability issues and propose solutions. Tables 2 and 3 shows the results of this evaluation, including heuristic categories, usability problems, and modifications to the software prior to user-based testing. For example, inspection of a patient Web-based interface revealed that small radio buttons for symptom scoring tended to be difficult to use by people with poor eyesight and limited dexterity. Thus, the buttons were made larger and the severity of symptom was included in the button (Figure 2). Heuristic and initial patient testing identified difficulty with the use of radio buttons to indicate response choices for symptom collection, lack of apparent progress indicators, and the size, color, and positioning of navigation buttons (forward and backward) as potential usability issues. Based on these findings, improvements were made to the interface, such as larger buttons, improved indication of button functions, and graphical and numerical progress indicators.

**Table 2:** Results of the heuristic analysis and resulting software solutions (patient).

Heuristic categories	Patient interface issue	Patient interface solution
Visibility of system status	Users cannot tell how many questions remain in a survey	Quantify number of pages or questions remaining and show progress
Match between the system and the real world^a^	Buttons are not representative of their function	Match the shape of buttons to function and add pictures to buttons
User control and freedom	Navigation to move backward and forward not clear	Optimize size, shape, location, and color of forward and backward buttons
Consistency and standards	Inconsistent labeling of PRO-CTCAE^b^ symptom terms	Present labeling in consistent format
Error prevention	Small buttons close together, which increases the risk of selecting the wrong button	Increase size, labeling, and spacing of buttons
Aesthetic and minimalist design	Too many radio buttons in variable positions	Enlarge or enhance appropriate buttons; avoid open spaces and scrolling
Help users recognize and recover from errors	No help available	Create help documentation^a^
Disability accommodations^c^	Radio buttons difficult to use; text too small	Create large target area for clicks and touch; make text larger and use easy-to-read font, and appropriate for color-blind individuals

^a^Defined as functionality intuitively matching the intended function.

^b^PRO-CTCAE: Patient-Reported Outcomes version of the Common Terminology Criteria.

^c^Item is not part of standard traditional heuristics and was added for the specific needs of our patient population.

**Table 3:** Results of the heuristic analysis and resulting software solutions (professional users).

Heuristic categories	Professional interface issue	Professional interface solutions
Visibility of system status	Users cannot tell during pauses if the system is processing a task or is frozen	Create a spinning icon to show when the system is processing a task
Match between the system and the real world^a^	Users cannot tell if the survey is ready for patients to complete; survey schedule presented as a list instead of the calendar	Add clear terms for functions (eg, “finalize” to finish a survey); add a graphical calendar to display or alter patient survey schedule
User control and freedom	No ability to customize interface	Provide ability for users to organize interface and modules that they use most often
Consistency and standards	Inconsistent labeling of PRO-CTCAE^b^symptom terms; no ability to download collected data in a standardized format	Present labeling in consistent format; enable data to be downloaded for analysis in common formats
Error prevention	Dates difficult to read, interpret, or change in the survey schedule	Present information in a clear calendar format
Recognition rather than recall	Software does not remember study number or site for a user; the user has to frequently re-enter same data	Software defaults study number and site for users once entered; software auto-populates user preferences or data
Flexibility and efficiency of use	No “dashboard” of essential or time-sensitive data	Create dashboard displaying key information and upcoming surveys
Aesthetic and minimalist design	Menu buttons are difficult to use	Make more functions easily available on the dashboard
Help users recognize and recover from errors	No explanations provided to users to understand causes of errors	Provide popup messages to help correct and prevent future errors

^a^Defined as functionality intuitively matching the intended function.

^b^PRO-CTCAE: Patient-Reported Outcomes version of the Common Terminology Criteria.

**Figure 3:**
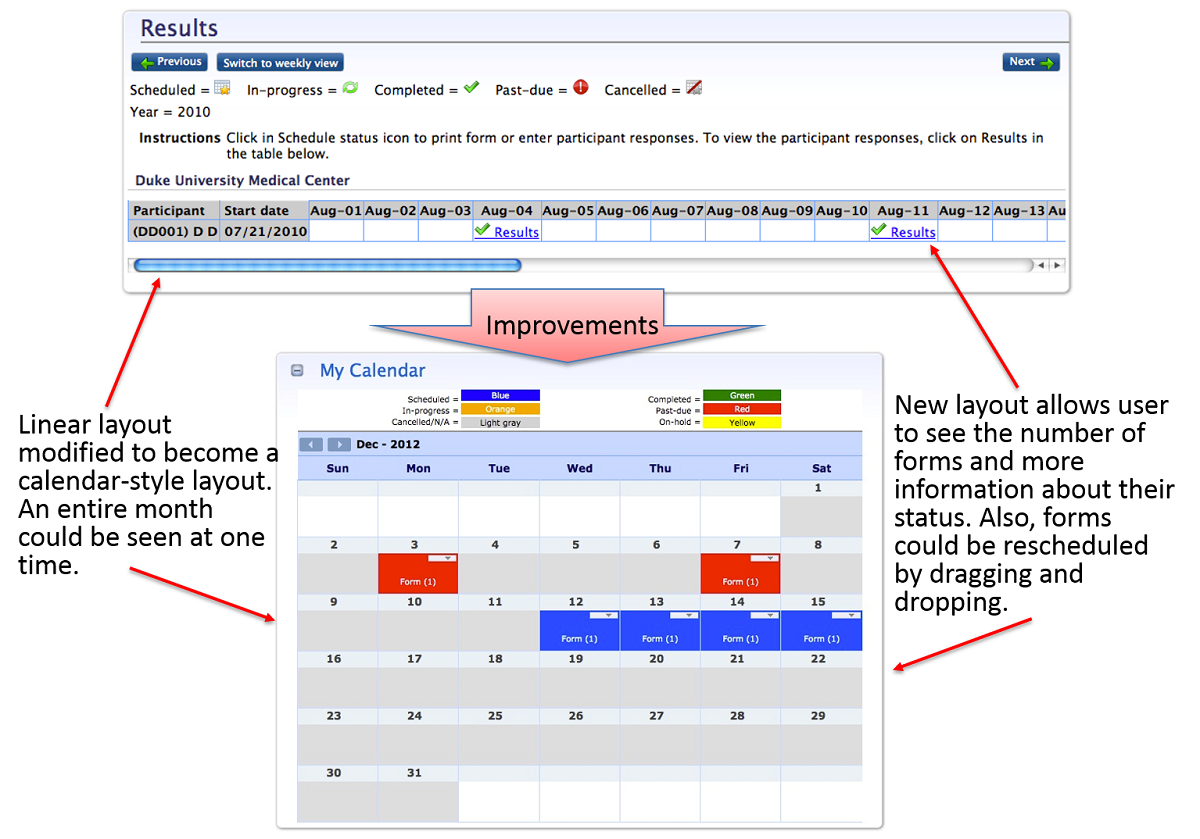
Screenshots of the Patient-Reported Outcomes version of the Common Terminology Criteria for Adverse Events (PRO-CTCAE) before and after usability improvements to benefit end-users: professional staff.

In the Web-based interface for professional staff, the survey scheduling function was modified from a horizontal list to a more intuitive calendar graphic (Figure 3). Testing with clinicians and research associates identified difficulties in setting and changing schedules for the survey administration to patients. This was improved for Round 2 with the addition of a calendar-type layout with drag-and-drop functionality that enabled survey schedules to be easily configured and modified at the patient level.

### Aim 2: User-Based Usability Assessment

#### Participants

Multimedia Appendix 1 shows the characteristics of patients and professionals (physicians, nurses, and research associates) who participated in the usability testing. A total of 169 patients participated; 54.4% (92/169) were females, 18.3% (31/169) were older than 65 years, 31.9% (54/169) were nonwhite, 26.0% (44/169) had high school education or less, and 18.3% (31/169) had limited prior computer experience. Next, 47 professional users participated, including 51% (24/47) clinicians and 49% (23/47) research associates. Clinicians comprised 26% (12/47) physicians and 26% (12/47) nurses.

#### Patient Usability Testing: Round 1

**Figure 4:**
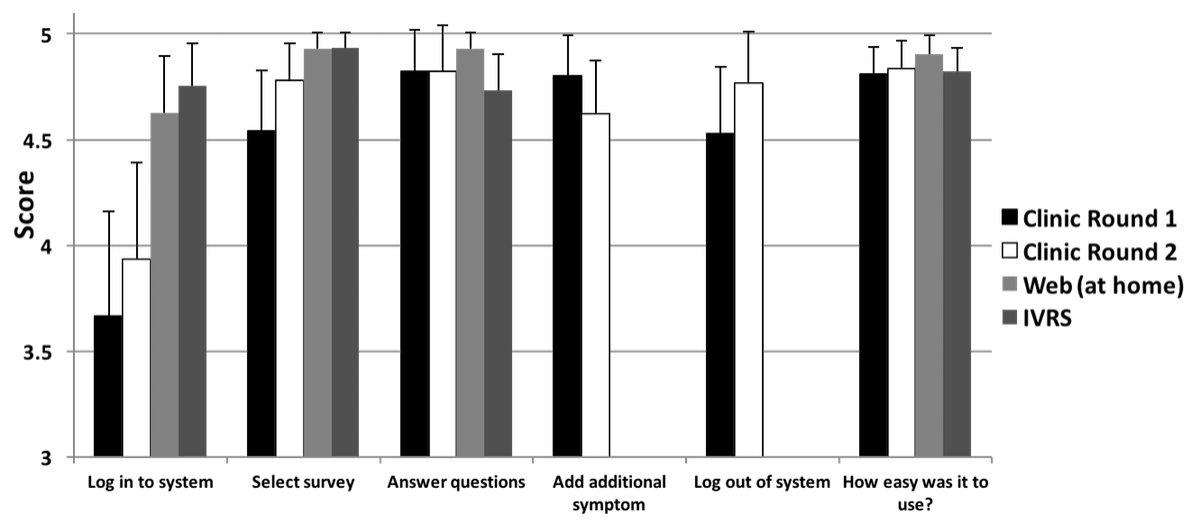
Task performance by patients using the 0-5 quantitative task completion scale (Table 1) and tasks from Multimedia Appendix 1. IVRS: interactive voice response system.

Round 1 testing identified favorable initial usability, with a mean SUS score of 86 for the patient Web-based interface (95% CI 83-90). Figure 4 shows the mean scores for each of the specific tasks using the task completion 0-5 scale. Across all tasks, the mean score was 4.47 (95% CI 4.31-4.62). The only task that was significantly more difficult compared to other tasks was logging into the system (task score 3.67; 95% CI 3.18-4.16; *P*<.001) as shown in Figure 4.

In addition, 51% (90/175) of the comments generated from the think-aloud procedure in Round 1 signified a positive appraisal of the system usability, despite using a protocol designed to find usability problems (Multimedia Appendix 1). Furthermore, 45.1% (79/175) of the patient comments identified areas for improvement, including difficulties with passwords, logging into the system, and problems with the standardized category “match between system and real world” (ie, the task does not intuitively match the intended function).

#### Professionals (Clinicians and Research Associates) Usability Testing: Round 1

**Figure 5:**
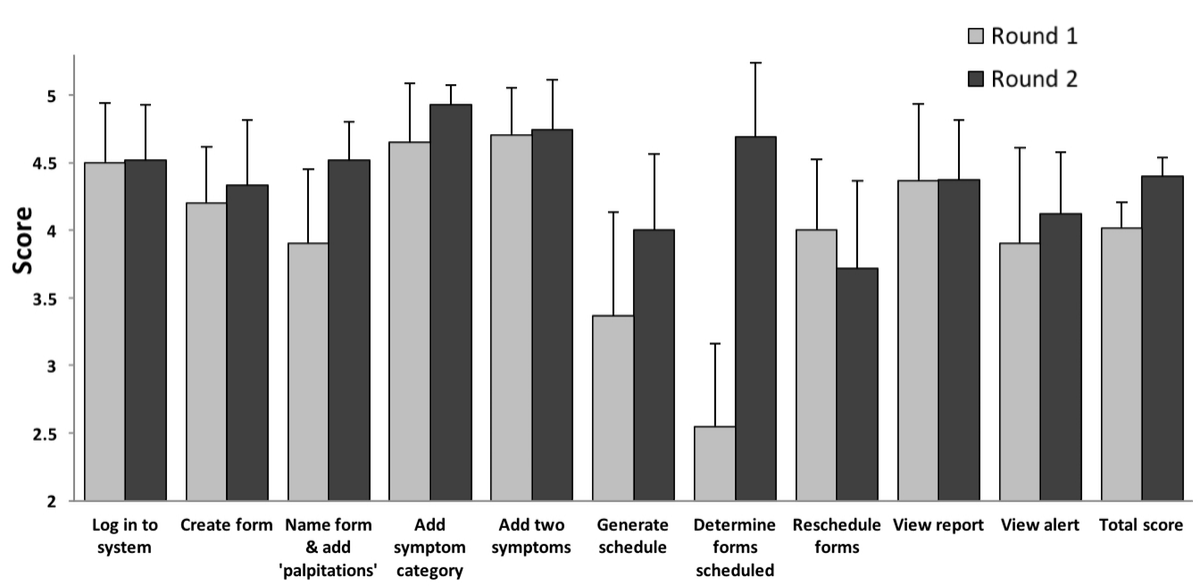
Task performance by professionals using the 0-5 quantitative task completion scale (Table 1) and tasks from Multimedia Appendix 1.

Overall, usability of the system based on the SUS score was 71 (95% CI 60-82). Figure 5 shows the mean task completion score for professional staff users. Moderate to high initial usability was seen across tasks with a mean score of 4.02 (95% CI 3.82-4.21).

Several tasks were identified by professional users as difficult or cumbersome, including determining the number of PRO surveys to be administered, monitoring patients’ completion of surveys, and creation of a schedule for survey administration. Determining the number of surveys to be scheduled was rated as significantly more difficult than other tasks (task score 2.55; 95% CI 1.90-3.20; *P=*.002), with a trend in task completion score indicating difficulty with scheduling the initial date for survey administration (task score 3.36; 95% CI 2.55-4.19; *P=*.08).

In Round 1 testing, professionals offered 141 comments about system usability and provided recommendations to the improve the flexibility and efficiency of use and to provide an aesthetic and minimalist design, recognition rather than recall, a match between the system and the real world (ie, the functionality intuitively matches the intended function), and consistency (Multimedia Appendix 1).

### Aim 3: System Improvements Between Rounds of Testing

Between rounds of user-based testing, software modifications were made based on study results. Specific improvements included functionality for remembering user preferences (eg, defaulting to a user’s institution, specific study number or name, and calendar preferences), minimizing the number of required clicks and dialog boxes, and simplifying the design to make the system more intuitive (Figure 3; Multimedia Appendix 1; specific example shown in Figure 2).

A major change to the clinician interface involved the inclusion of a calendar view for PRO-CTCAE survey scheduling. This calendar view could also simultaneously display scheduled surveys for multiple participants on the same day (Figure 3). Other significant changes included the creation of a “dashboard”-type screen upon login, which displayed clinical alerts, upcoming surveys, and the monthly calendar of surveys.

#### Patient Usability Testing: Round 2

In Round 2, usability remained high with a mean SUS score of 82 (95% CI 76-88) for the patient Web-based interface as tested in the clinic compared with a mean score of 86 (*P=*.22 for comparison) in Round 1. Participants who tested the Web-based interface or IVR system remotely and without staff assistance provided mean SUS scores of 92 (95% CI 88-95) for the home Web-based interface and 89 (95% CI 83-95) for the IVR system.

Task completion scores were also high with average score of 4.58 (95% CI 4.45-4.72) for the patient Web-based interface testing in the clinic, 4.85 (95% CI 4.77-4.93) for remote Web testing, and 4.74 (95% CI 4.66-4.82) for remote IVR system testing (Figure 4). Notably, logging into the system continued to be documented as a significant problem when using the patient interface in the clinic where internet connections were inconsistent; the mean score for the task of logging into the system was significantly lower than that for other tasks (3.93: 95% CI 3.46-4.41; *P=*.001). The scores for the remainder of tasks were not found to be markedly different, and the presence of consistently high scores across tasks suggested a ceiling effect.

We analyzed patient user comments separately for clinic-based versus remote use and classified them thematically (Multimedia Appendix 1). The most common theme was “difficulty in logging into the system,” which substantially improved between Rounds 1 and 2 (2.3% in Round 2 vs 9.1% of comments in Round 1). The second-most common critique was a lack of “match between the system and real world” (ie, functionality not intuitively matching the intended function), and this mismatch decreased after Round 2 testing (1.7% vs 8.6%). The IVR system component of the PRO-CTCAE system generated negative comments regarding “visibility of system status” (3.7%) and “flexibility and efficiency of use” (2.0%). Based on these results, we concluded that a satisfactory level of patient usability had been attained.

#### Professional Staff Usability Testing: Round 2

Round 2 testing with professional staff focused on specific usability issues that had been identified in Round 1 and modified through software modifications. In Round 2, the SUS score was 75 (95% CI 69-82), compared with the Round 1 score of 71 (*P=*.47 for comparison). Across all tasks, the mean task performance score was 4.40 (95% CI 4.26-4.54), which was significantly improved from Round 1 (vs 4.02, 95% CI 3.82-4.21, *P=*.001). Usability scores improved for the 2 tasks with marked difficulty in Round 1, specifically, “determining the number of surveys to be scheduled” (improved from 2.55 in Round 1 to 4.69, 95% CI 4.14-5.24, *P*<.001) and “creating an initial survey administration schedule” (improved from 3.36 in Round 1 to 4.00, 95% CI 3.44-4.56, *P=*.19). Furthermore, the task of “naming a form and adding a symptomatic toxicity” significantly improved from 3.90 in Round 1 to 4.52 (95% CI 4.24-4.80; *P=*.04).

Compared with Round 1, professionals offered fewer negative comments regarding “aesthetic and minimalist design,” as well as “match between the system and the real world” (Multimedia Appendix 1). Negative comments persisted in the heuristic domains of “recognition rather than recall” and “flexibility and efficiency of use.” The investigative team discussed these comments and concluded that they were consistent with the learning curve typically associated with the use of any complex software and that further modifications to address these comments were unlikely to improve usability of the system.

## Discussion

A rigorous usability evaluation of a software system for the PRO-CTCAE survey administration, using heuristic and user-based testing with 169 patients and 47 staff members, with iterative modifications between rounds of testing, yielded a highly usable system for electronic capture of PRO-CTCAE responses. As the system for survey scheduling and administration must be integrated into the complex workflow of cancer clinical trials, comprehensive usability testing by both patients and professional staff was essential. In comparison to many usability assessments, this study included a relatively large and diverse sample that included patients, clinicians, and research associates as users. Moreover, a purposeful enrollment strategy to achieve a patient sample that was diverse with respect to age, ethnicity, educational attainment, and digital literacy strengthens the generalizability of our results.

Based on these favorable usability outcomes, the PRO-CTCAE software system has been implemented in 5 large, multicenter cancer clinical trials in the NCI National Clinical Trials Network and the NCI Community Oncology Research Program (NCORP; NCT01515787, NCT02037529, NCT02414646, NCT01262560, NCT02158637). These findings have also informed the specification of the required functionalities for a downloadable mobile app to collect PRO-CTCAE data within the Medidata Rave clinical trials data management system, thereby supporting the inclusion of PRO-CTCAE in numerous NCI-sponsored cooperative group trials.

This study has several limitations, which should be considered while interpreting the results of this study. First, the system was only assessed in outpatients, and therefore, it is not known whether comparable usability would be seen in hospitalized patients. Second, the sampling did not include participants with visual, auditory, or tactile impairments that might restrict their use of computer hardware or a telephone-based IVR system use of hardware. Finally, we did not enrich our sample for participants with performance status impairment, and approximately 20% of patients enrolled were older than 65 years and had lower digital literacy.

In conclusion, heuristic evaluation followed by iterative rounds of multistakeholder user-based testing and refinement evolved the PRO-CTCAE software into an effective and well-accepted platform for patient-reporting of symptomatic AEs in cancer clinical trials.

## Appendix

Multimedia Appendix 1 Information on usability domains used to define the PRO-CTCAE system; tasks included in the evaluation of the patient portal and professional interface; patient user demographics; professional user demographics; patients’ qualitative comments about the system; professionals’ qualitative comments about the system.

Multimedia Appendix 2 Professional and patient think-aloud protocols.

## Abbreviations

AE: adverse event
ANOVA: analysis of variance
CTCAE: Common Terminology Criteria for Adverse Events
IVR: interactive voice response
IVRS: interactive voice response system
NCI: National Cancer Institute
PRO-CTCAE: Patient-Reported Outcomes version of the Common Terminology Criteria for Adverse Events
SUS: System Usability Scale
