# Supplementary material for: Software for Administering the National Cancer Institute’s Patient-Reported Outcomes Version of the Common Terminology Criteria for Adverse Events: Usability Study
Source: JMIR Hum Factors. 2018 Jul 16;5(3):e10070. doi: 10.2196/10070 (PMC6066634; doi:10.2196/10070)
Supplement: Multimedia Appendix 1 [file humanfactors_v5i3e10070_app1.pdf]

APPENDICES/SUPPLEMENTAL MATERIAL FOR:

Usability testing of software for administering the National Cancer Institute's Patient-Reported Outcomes version of the Common Terminology Criteria for Adverse Events (PRO-CTCAE)

Martin W. Schoen, MD, MPH, Ethan Basch, MD, MSc, Lori L. Hudson, PhD, Arlene E. Chung, MD, MHA, MMCi, Tito R. Mendoza PhD, Sandra A. Mitchell PhD, CNRP, Diane St. Germain, RN, MS, Paul Baumgartner, MS, Laura Sit, MS, Lauren J. Rogak, MA, Marwan Shouery, MS, Eve Shalley, BA, Bryce B. Reeve, PhD, Maria R. Fawzy, MHA, Nrupen A. Bhavsar, PhD, Charles Cleeland, PhD, Deborah Schrag, MD, MPH, Amylou C. Dueck, PhD, Amy P. Abernethy, MD, PhD

Correspondence to:
Ethan Basch, MD
University of North Carolina
170 Manning Drive
Chapel Hill, NC 27516
ebasch@med.unc.edu
919-843-3055

Multimedia Appendices

eTable 1 – Usability domains used to define the PRO-CTCAE system

Usability Domains	Definition
Ease of learning	How fast can someone unfamiliar with the user interface learn it sufficiently well to accomplish basic tasks?
Efficiency of use	How fast can an experienced user accomplish tasks?
Memorability	If someone has used the system before, can they remember enough to use it effectively the next time or do they have to relearn some or all of its components?
Error frequency and severity	How often do users make errors while using the system, how serious are these errors, and how do users recover from these errors?
Subjective satisfaction	How much does the user like using the system?

eTable 2. Tasks included in the evaluation of the patient portal and professional interface.

Task	Description
**Patient System**	
Log into system	Log into system as a patient
Select Survey	Select a survey to take as a patient
Answer survey questions	Answer several survey questions, including conditional branching
Add additional symptom	Add an additional symptom that a patient could experience
Log out of system	Log out of the system
**Professional System**	
Log into system	Log into system as a professional
Create Form	Create a survey form
Name Form & add “palpitations”	Name form and add the symptom “palpitations”
Add category of symptoms	Add a separate category of symptoms
Add two additional symptoms	Add additional symptoms to survey
Generate schedule for form	Generate a schedule for form administration
Monitor Forms	Monitor the form administration to patient users
Determine the number of forms scheduled	Determine the number of forms that had been scheduled for a patient used
How Easy Was it to Reschedule?	Report user ease with rescheduling forms for a patient user

View Report	View reports generated from patient user responses
View Alert	View clinical alert that was triggered by a patient
Clear Alert	Clear and managed an alert that was triggered

eTable 3. Patient user demographics.

**Characteristics**		**Clinic-Based Testing**		**Remote Testing**		**Total n=169 n (%)**
		**Round 1 n=36 n (%)**	**Round 2 n=44 n (%)**	**Web-based interface n=44 n (%)**	**IVR system n=45 n (%)**	
**Age (years)**						
	Mean (SD)	51.6 (4.6)	53.8 (3.7)	51.3 (3.9)	55.4 (4.1)	53.0 (2.0)
	>65 years old	6 (17)	8 (18)	7 (16)	10 (22)	31 (18)
**Sex**						
	Female	21 (59)	23 (52)	23 (52)	25 (57)	92 (54)
**Race**						
	White	29 (81)	28 (64)	29 (66)	28 (62)	114 (67)
	Asian	1 (3)	2 (5)	3 (70)	0 (0)	6 (4)
	Black	3 (8)	10 (23)	11 (25)	17 (38)	41 (24)
	Other/multiple race	3 (8)	3 (7)	1 (2)	0 (0)	7 (4)
**Ethnicity**						
	Hispanic	3 (8)	5 (11)	6 (14)	3 (7)	17 (10)
**Education**						
	High School or Less	7 (19)	11(25)	11 (25)	15 (33)	44 (26)
	Some College or College Degree/ Equivalent	19 (53)	25 (59)	25 (57)	22 (49)	91 (54)
	Graduate Education	10 (27)	7 (16)	8 (17)	8 (18)	33 (20)
**Computer use**						
	Not comfortable or rarely use computers	6 (17)	8 (19)	6 (14)	11 (24)	31 (19)
	Comfortable checking email	6 (17)	9 (21)	7 (16)	15 (33)	37 (22)
	Regularly use computers	24 (66)	26 (60)	31 (70)	19 (42)	100 (59)

eTable 4. Professional user demographics.

**Characteristics**		**Round 1 n=20 n (%)**	**Round 2 n=27 n (%)**	**Total n=47 n (%)**
**Age**				
	Mean (SD)	39.6 (6.5)	35 (4.3)	37 (5.2)
**Sex**				
	Female	16 (80)	19 (70)	35 (74)
**Race**				
	White	14 (70)	18 (67)	32 (68)
	Asian	4 (20)	5 (19)	9 (19)
	Black	0 (0)	3 (11)	3 (6)
	Other/multiple race	2 (10)	1 (4)	3 (6)
**Ethnicity**				
	Hispanic	2 (10)	1 (4)	3 (6)
**Education**				
	High School Graduate	0 (0)	1 (4)	1 (2)
	Some College or College Degree	9 (45)	10 (37)	9 (40)
	Graduate Education	11 (55)	16 (59)	25 (57)
**Computer use**				
	Not comfortable or rarely use computers	0 (0)	0 (0)	0 (0)
	Comfortable checking email	0 (0)	0 (0)	0 (0)
	Regularly use computer	20 (100)	27 (100)	47 (100)
**Professional role**				
	Research Associate	6 (30)	17 (63)	23 (49)
	Nurse	7 (35)	5 (19)	12 (26)
	Physician	7 (35)	5 (19)	12 (26)

eTable 5. Patients' qualitative comments about the system.

Comment Categories	Clinic-based Testing		Remote Testing		Total Patients
	Round 1 n=36 n (%)	Round 2 n=44 n (%)	Web-based interface n=44 n (%)	IVR system n=45 n (%)	n=169 n (%)
Total comments	175	303	302	407	1187
Positive comments	90 (51)	217 (71)	230 (76)	302 (74)	839 (71)
Neutral comments	6 (4)	35 (12)	37 (12)	39 (10)	117 (10)
Negative comments	79 (45)	51 (17)	35 (12)	66 (16)	231 (19)
**Negative Comment Categories**					
Visibility of system status	6 (3.4)	1 (0.3)	2 (0.7)	15 (3.7)	24 (2.0)
Match between the system and the real world[a]	15 (8.6)	6 (2.0)	4 (1.3)	7 (1.7)	32 (2.7)
User control and freedom	2 (1.1)	2 (0.6)	2 (0.7)	9 (2.1)	15 (1.3)
Consistency and standards	3 (1.7)	4 (1.3)	1 (0.3)	1 (0.3)	9 (0.8)
Flexibility and efficiency of use	4 (2.9)	2 (0.7)	1 (0.3)	8 (2.0)	16 (1.4)
Aesthetic and minimalist design	3 (1.7)	5 (1.7)	0 (0)	0 (0)	8 (0.7)
Disability accommodation	6 (3.4)	10 (3.3)	4 (1.3)	5 (1.2)	25 (2.1)
Password problem	2 (1.1)	1 (0.3)	2 (0.7)	4 (1.0)	9 (0.8)
Difficulty finding site, URL problem, browser error	16 (9.1)	5 (1.7)	8 (2.7)	NA	29 (2.4)
User error	8 (4.6)	6 (2.0)	3(1.0)	2 (0.5)	19 (1.6)

[a]Defined as functionality intuitively matching the intended function.

eTable 6 Professionals' qualitative comments about the system

Comment Categories	Round 1<br>n= 20<br>n (%)	Round 2<br>n= 27	Total<br>n= 47
Total Comments	141	111	252
**Negative comment categories**			
Visibility of system status	2 (1.4)	5 (4.5)	7 (2.8)
Match between the system and the real world[a]	20 (14)	6 (5.4)	26 (10)
User control and freedom	12 (8.5)	4 (3.6)	16 (6.3)
Consistency and standards	17 (12)	19 (17.1)	36 (14)
Error prevention	6 (4.3)	10 (9.0)	16 (6)
Recognition rather than recall	22 (16)	12 (11)	34 (14)
Flexibility and efficiency of use	25 (18)	26 (23)	51 (20)
Aesthetic and minimalist design	24 (17)	12 (11)	26 (14)
Help users recognize, diagnose, and recover from errors	4 (2.8)	1 (0.9)	5 (2.0)
Help and documentation	3 (2.1)	10 (9.0)	13 (5.2)
Password problem	1 (0.7)	4 (3.6)	5 (2.0)
Difficulty finding site, URL problem, browser error	3 (2.1)	1 (0.9)	4 (1.6)
User error	1 (0.7)	1 (0.9)	2 (0.8)

[a]*Defined as functionality intuitively matching the intended function.*
