# Supplementary material for: Software for Administering the National Cancer Institute’s Patient-Reported Outcomes Version of the Common Terminology Criteria for Adverse Events: Usability Study
Source: JMIR Hum Factors. 2018 Jul 16;5(3):e10070. doi: 10.2196/10070 (PMC6066634; doi:10.2196/10070)
Supplement: Multimedia Appendix 2 [file humanfactors_v5i3e10070_app2.pdf]

Prompts Clinical User

SiteID_ParticipantID (eg. Duke_001 or MDA_001 or MSKCC_001) ______________________________

Tester ID ______________________________

1.Please open your browser and point it to http://proctcae.nci.nih.gov

- ☐ Internet Explorer
- ☐ Safari
- ☐ Firefox
- ☐ Chrome
- ☐ Other

Question 1 other specify ______________________________

2.Please use Internet Explorer and open http://proctcae.nci.nih.gov

- ☐ 0-Task not completed...
- ☐ 1-Task performed after multiple prompts and/or help
- ☐ 2-Completed with single prompt
- ☐ 3-Achieved task with confusion or with multiple inappropriate clicks
- ☐ 4-Task performed with hesitation or single error
- ☐ 5-Completed task easily

Question 2 comments

3.What is your initial impression of the home page?

4.There are various studies that utilize this software. The program allows you to create questionnaires that ask patients about any symptoms they might be having while receiving treatment. Please create a form for the study _______________

- ☐ 0-Task not completed...
- ☐ 1-Task performed after multiple prompts and/or help
- ☐ 2-Completed with single prompt
- ☐ 3-Achieved task with confusion or with multiple inappropriate clicks
- ☐ 4-Task performed with hesitation or single error
- ☐ 5-Completed task easily

Question 4 comments

5. Name this form _________. The form will need to assess these symptoms. a. Palpitations a. Palpitations

- ☐ 0-Task not completed...
- ☐ 1-Task performed after multiple prompts and/or help
- ☐ 2-Completed with single prompt
- ☐ 3-Achieved task with confusion or with multiple inappropriate clicks
- ☐ 4-Task performed with hesitation or single error
- ☐ 5-Completed task easily

5. b. All eye disorders

- ☐ 0-Task not completed...
- ☐ 1-Task performed after multiple prompts and/or help
- ☐ 2-Completed with single prompt
- ☐ 3-Achieved task with confusion or with multiple inappropriate clicks
- ☐ 4-Task performed with hesitation or single error
- ☐ 5-Completed task easily

5.c. two additional symptoms of your choice

- ☐ 0-Task not completed...
- ☐ 1-Task performed after multiple prompts and/or help
- ☐ 2-Completed with single prompt
- ☐ 3-Achieved task with confusion or with multiple inappropriate clicks
- ☐ 4-Task performed with hesitation or single error
- ☐ 5-Completed task easily

Question 5 comments

6.Would it be helpful to have groups of common symptoms or disease-specific question sets in building these forms?

- ☐ No
- ☐ Yes
- ☐ conditional
- ☐ Explanation

Question 6 comments

Great, now please advance to the next screen

7.Now, you will generate a schedule for this form. The schedule should have the participant fill it out on day 1 of each 21 day cycle, for 4 cycles.

- ☐ 0-Task not completed...
- ☐ 1-Task performed after multiple prompts and/or help
- ☐ 2-Completed with single prompt
- ☐ 3-Achieved task with confusion or with multiple inappropriate clicks
- ☐ 4-Task performed with hesitation or single error
- ☐ 5-Completed task easily

Question 7 comments

8. Future versions of this software would allow you to create scheduling templates and integrate the patient

9.a. palpitations or blurry vision

- ☐ 0-Task not completed...
- ☐ 1-Task performed after multiple prompts and/or help
- ☐ 2-Completed with single prompt
- ☐ 3-Achieved task with confusion or with multiple inappropriate clicks
- ☐ 4-Task performed with hesitation or single error
- ☐ 5-Completed task easily

9.b. with a frequency greater than or equal to

- ☐ 0-Task not completed...
- ☐ 1-Task performed after multiple prompts and/or help
- ☐ 2-Completed with single prompt
- ☐ 3-Achieved task with confusion or with multiple inappropriate clicks
- ☐ 4-Task performed with hesitation or single error
- ☐ 5-Completed task easily

9.c.This form should notify the treating physician, site CRA and nurse

- ☐ 0-Task not completed...
- ☐ 1-Task performed after multiple prompts and/or help
- ☐ 2-Completed with single prompt
- ☐ 3-Achieved task with confusion or with multiple inappropriate clicks
- ☐ 4-Task performed with hesitation or single error
- ☐ 5-Completed task easily

Question 9 comments

10.This is a multi-site study, should local sites be able to change the notification scheme?

- ☐ No
- ☐ Yes

11.What would be your preference on when and how to receive notifications? As a (type of clinical provider, MD, nurse, etc), what are your thoughts on this function? Would you want these notifications sent to the Cancer Adverse Event Reporting System (caAERS)?

12.Great please save and continue to the next screen. Now review the questions. Is it easy to review the questions and determine if you included all the items of interest?

13.Now we are going to move on to a different set of tasks, which involve monitoring forms. ..

- ☐ 0-Task not completed...
- ☐ 1-Task performed after multiple prompts and/or help
- ☐ 2-Completed with single prompt
- ☐ 3-Achieved task with confusion or with multiple inappropriate clicks
- ☐ 4-Task performed with hesitation or single error
- ☐ 5-Completed task easily

Question 13 comments

14. a.How many forms are scheduled for the participant this month?

- ☐ 0-Task not completed...
- ☐ 1-Task performed after multiple prompts and/or help
- ☐ 2-Completed with single prompt
- ☐ 3-Achieved task with confusion or with multiple inappropriate clicks
- ☐ 4-Task performed with hesitation or single error
- ☐ 5-Completed task easily

14. b. How many forms are scheduled for the participant complete next month?

- ☐ 0-Task not completed...
- ☐ 1-Task performed after multiple prompts and/or help
- ☐ 2-Completed with single prompt
- ☐ 3-Achieved task with confusion or with multiple inappropriate clicks
- ☐ 4-Task performed with hesitation or single error
- ☐ 5-Completed task easily

Question 14 comments

15. Sometimes a form

- ☐ 0-Task not completed...
- ☐ 1-Task performed after multiple prompts and/or help
- ☐ 2-Completed with single prompt
- ☐ 3-Achieved task with confusion or with multiple inappropriate clicks
- ☐ 4-Task performed with hesitation or single error
- ☐ 5-Completed task easily

Question 15 comments

16. a. Next, add a new subject to the _(site Usability)_ study.

- ☐ 0-Task not completed...
- ☐ 1-Task performed after multiple prompts and/or help
- ☐ 2-Completed with single prompt
- ☐ 3-Achieved task with confusion or with multiple inappropriate clicks
- ☐ 4-Task performed with hesitation or single error
- ☐ 5-Completed task easily

16. Attempts ________________________________

16.b. Are there any fields that you think should be added or removed from this section?

17.Please choose to change the notification scheme or not and select

- ☐ 0-Task not completed...
- ☐ 1-Task performed after multiple prompts and/or help
- ☐ 2-Completed with single prompt
- ☐ 3-Achieved task with confusion or with multiple inappropriate clicks
- ☐ 4-Task performed with hesitation or single error
- ☐ 5-Completed task easily

Question 17 comments

18.Now verify that this person was added

- ☐ 0-Task not completed...
- ☐ 1-Task performed after multiple prompts and/or help
- ☐ 2-Completed with single prompt
- ☐ 3-Achieved task with confusion or with multiple inappropriate clicks
- ☐ 4-Task performed with hesitation or single error
- ☐ 5-Completed task easily

Question 18 comments

19.Now we are going to look at reports for a form that has already been filled out. Subject _(A_A)_ has completed a form called _(Sample Data)_

- ☐ 0-Task not completed...
- ☐ 1-Task performed after multiple prompts and/or help
- ☐ 2-Completed with single prompt
- ☐ 3-Achieved task with confusion or with multiple inappropriate clicks
- ☐ 4-Task performed with hesitation or single error
- ☐ 5-Completed task easily

Question 19 comments

20. Please pull up a graph for the symptom

- ☐ 0-Task not completed...
- ☐ 1-Task performed after multiple prompts and/or help
- ☐ 2-Completed with single prompt
- ☐ 3-Achieved task with confusion or with multiple inappropriate clicks
- ☐ 4-Task performed with hesitation or single error
- ☐ 5-Completed task easily

Question 20 comments

How is the symptom

21.a.Overall study report. What do you think of this report?

- ☐ 0-Task not completed...
- ☐ 1-Task performed after multiple prompts and/or help
- ☐ 2-Completed with single prompt
- ☐ 3-Achieved task with confusion or with multiple inappropriate clicks
- ☐ 4-Task performed with hesitation or single error
- ☐ 5-Completed task easily

Question 21 a. comments

21.b. Symptom summary report. Is this useful for data analysis?

- ☐ 0-Task not completed...
- ☐ 1-Task performed after multiple prompts and/or help
- ☐ 2-Completed with single prompt
- ☐ 3-Achieved task with confusion or with multiple inappropriate clicks
- ☐ 4-Task performed with hesitation or single error
- ☐ 5-Completed task easily

Question 21 b. comments

22.Now let

- ☐ 0-Task not completed...
- ☐ 1-Task performed after multiple prompts and/or help
- ☐ 2-Completed with single prompt
- ☐ 3-Achieved task with confusion or with multiple inappropriate clicks
- ☐ 4-Task performed with hesitation or single error
- ☐ 5-Completed task easily

Question 22 comments

23.Now we are going to move on to a different set of tasks. Please return to the home page.

- ☐ 0-Task not completed...
- ☐ 1-Task performed after multiple prompts and/or help
- ☐ 2-Completed with single prompt
- ☐ 3-Achieved task with confusion or with multiple inappropriate clicks
- ☐ 4-Task performed with hesitation or single error
- ☐ 5-Completed task easily

24.You may recall that we set up

- ☐ 0-Task not completed...
- ☐ 1-Task performed after multiple prompts and/or help
- ☐ 2-Completed with single prompt
- ☐ 3-Achieved task with confusion or with multiple inappropriate clicks
- ☐ 4-Task performed with hesitation or single error
- ☐ 5-Completed task easily

Note how participant accesses the alerts (circle one):

- ☐ Date
- ☐ Participant ID
- ☐ Actions

Question 24 comments

25. Once you are finished reviewing them, clear the alerts and return to the home page.

- ☐ 0-Task not completed...
- ☐ 1-Task performed after multiple prompts and/or help
- ☐ 2-Completed with single prompt
- ☐ 3-Achieved task with confusion or with multiple inappropriate clicks
- ☐ 4-Task performed with hesitation or single error
- ☐ 5-Completed task easily

Question 25 comments

26.What are your thoughts about how these alerts are displayed? Are they useful?

27.This portion of the usability testing is complete. We really appreciate your willingness to use this software and share your thoughts about it.

Form Status

Complete?

- ☐ Incomplete
- ☐ Unverified
- ☐ Complete

Prompts Patient User

SiteID_ParticipantID (eg. Duke_001 or MDA_001 or MSKCC_001) __________________________________

Tester ID __________________________________

1.Please open your browser and point it to http://proctcae.nci.nih.gov

- ☐ Internet Explorer
- ☐ Safari
- ☐ Firefox
- ☐ Chrome
- ☐ Other

Question 1 other specify __________________________________

2.Please use Internet Explorer and open http://proctcae.nci.nih.gov Please log into the system.

- ☐ 0-Task not completed...
- ☐ 1-Task performed after multiple prompts and/or help
- ☐ 2-Completed with single prompt
- ☐ 3-Achieved task with confusion or with multiple inappropriate clicks
- ☐ 4-Task performed with hesitation or single error
- ☐ 5-Completed task easily

Question 2 comments

3.This software is designed for patients to report their symptoms over a period of time. Please try out the software; your answers can be whatever you like and will not be communicated to your medical team. This is for testing purposes only.

4.What is your initial impression of the home page?

5.What would be your preference on when and how to receive notifications that you have a symptom survey ready to be filled out (email, telephone call, in clinic)?

6.Please select the survey that is scheduled to fill out.

- ☐ 0-Task not completed...
- ☐ 1-Task performed after multiple prompts and/or help
- ☐ 2-Completed with single prompt
- ☐ 3-Achieved task with confusion or with multiple inappropriate clicks
- ☐ 4-Task performed with hesitation or single error
- ☐ 5-Completed task easily

Question 6 comments

7. Is it obvious where you would need to click to open the form you are scheduled to fill out?

- ☐ No
- ☐ Yes

Question 7 Explanation

8. Great. Now you will just answer the questions as they appear.

- ☐ 0-Task not completed...
- ☐ 1-Task performed after multiple prompts and/or help
- ☐ 2-Completed with single prompt
- ☐ 3-Achieved task with confusion or with multiple inappropriate clicks
- ☐ 4-Task performed with hesitation or single error
- ☐ 5-Completed task easily

Question 8 comments

9.What are your thoughts about how the questions are presented?

10.Are you aware of your progress through these questions?

11.You now have the option to add any additional symptoms of your choice. Please choose one from the list or enter one of your choice in the box

- ☐ 0-Task not completed...
- ☐ 1-Task performed after multiple prompts and/or help
- ☐ 2-Completed with single prompt
- ☐ 3-Achieved task with confusion or with multiple inappropriate clicks
- ☐ 4-Task performed with hesitation or single error
- ☐ 5-Completed task easily

Question 11 comments

12. Great, please continue then submit the study, return to the home page, and log out of the system

- ☐ 0-Task not completed...
- ☐ 1-Task performed after multiple prompts and/or help
- ☐ 2-Completed with single prompt
- ☐ 3-Achieved task with confusion or with multiple inappropriate clicks
- ☐ 4-Task performed with hesitation or single error
- ☐ 5-Completed task easily

13. This portion of the usability testing is complete. We really appreciate your willingness to use this software and share your thoughts about it.

Form Status

Complete?

- ☐ Incomplete
- ☐ Unverified
- ☐ Complete
